# A Multidimensional Approach of Surgical Mortality Assessment and Stratification (Smatt Score)

**DOI:** 10.1038/s41598-020-67164-6

**Published:** 2020-07-03

**Authors:** Sara Cutti, Catherine Klersy, Valentina Favalli, Lorenzo Cobianchi, Alba Muzzi, Marco Rettani, Guido Tavazzi, Maria Paola Delmonte, Andrea Peloso, Eloisa Arbustini, Carlo Marena

**Affiliations:** 1 Medical Direction, Foundation IRCCS San Matteo Hospital, Viale Golgi 19, 27100 Pavia, Italy; 2 Service of Clinical Epidemiology & Biometry, Foundation IRCCS San Matteo Hospital, Viale Golgi 19, 27100 Pavia, Italy; 3 Transplant Research Area, Foundation IRCCS San Matteo Hospital, Viale Golgi 19, 27100 Pavia, Italy; 4 General Surgery, Foundation IRCCS San Matteo Hospital, Viale Golgi 19, 27100 Pavia, Italy; 5 University of Pavia, Department of Clinical, Surgical, Diagnostic and Pediatric Sciences, Foundation IRCCS San Matteo Hospital, Viale Golgi 19, 27100 Pavia, Italy; 6 Department of Anesthesia and Intensive Care, Foundation IRCCS San Matteo Hospital, Viale Golgi 19, 27100 Pavia, Italy

**Keywords:** Epidemiology, Risk factors

## Abstract

Surgical mortality is the most significant measure of outcome in surgical healthcare. The objective was to assess surgical 30 days mortality and improve the identification of predictors for personalized risk stratification of patients undergoing elective and emergency surgery. The study was conducted as a single-center cohort retrospective observational study, based on the analysis of data collected from patients surgically treated from 2002 to 2014 in a multi-disciplinary research and care referral hospital with global case mix of 1.27. The overall in-hospital mortality rate was 1.89% (95% CI 1.82–1.95). In the univariable analysis, numerous predictors were significantly associated with in-hospital death following surgery. In the multivariable model, age, BMI (Body Mass Index), ASA score, department, planned surgical complexity, surgical priority, previous surgeries in the same hospitalization, cardiovascular, pulmonary, hepato-renal comorbidities, drug intolerance, cancer and AIDS were independently associated with mortality after surgery. At logistic regression, the computed SMATT score (graded 0–100), generated on the basis of multivariate analysis, demonstrated a good discrimination (10-fold cross-validated AUC-ROC 0.945, 95%CI 0.941–0.948) and correctly classified 98.5% of those admissions with a probability of death >50%. The novel SMATT score, based on individual preoperative and surgical factors, accurately predicts mortality and provides dynamic information of the risk in redo/reoperative surgery.

## Introduction

Mortality is one of the most important outcomes for surgical procedures and one this easily defined, at least during a patient admission episode. However, perioperative mortality rates in themselves are difficult to interpret without risk stratification^1,2^. An accurate risk stratification tool is a key-player in the perioperative diagnostic/therapeutic pathway and enables meaningful comparison of surgical outcomes by adjusting for specific risk^3^. Almost 313 million surgical procedures are performed each year worldwide^4,5^, with at least 77,2 million disability-adjusted life-years (DALYs) avoidable by basic, life-saving surgical care^6^. Surgical needs account for 28–32% of the overall global burden of diseases: surgical activity and complexity will further increase in the near future mainly due to higher life expectancy and increase of chronic comorbidities^7,8^. In 2050, 16% of the world’s population will be older than 65 years, with ageing impacting surgical activity^9^. In this demographic transition, correct estimates of comorbidities and risk factors are expected to play a role in efficient decision-making. Technology advancements (Intensive Care Unit, minimally invasive surgery, robotics, hybrid interventions, and advanced anesthesiology procedures) make almost every patient fit-for-surgery^10^. Benchmarking of surgical outcomes across hospitals can help refining standards for estimating surgical risk but possible advantages are limited by different methods adopted for risk stratification and require appropriate adjustment models because postoperative inpatient mortality reflects individual risk factors, surgery complexity and data collection^11^.

Different scoring systems exist and are currently used both in clinical and perioperative settings (see Table 1)^12–18^, each considering different sets of pre-operative predictors. A predictive score should have some mandatory features: feasibility at the bedside, reproducibility and reliability. Moreover, a perioperative score should be able to stratify the risk according to different surgical specialties.

**Table 1:** Factors included in the different risk scores: ■factor shared in the different scores; ◯ uniquely present; ◆ included in comorbidities.

Mortality Score	POSSUM	APACHE II	Charlson	SORT	SRS -NSQIP	NELA	SMATT
Age	■	■	■	■	■	■	■
Gender					■	■	■
Present smoker					◯		
Functional Status					◯		
BMI					◯		■
Surgical procedure				■	■		
Grade of surgery	■			■		■	■
N° of procedure	■					■	■
Urgency of surgery	■			■	■	■	■
Surgical specialty							■
ASA score				■	■	■	■
Cirrhosis		■					◆
Heart failure	■	■			■	■	◆
COPD	■	■	■		■	■	◆
Hypertension					◯		◆
Acute Renal Failure		■			■		◆
Chronic Renal Failure (dialysis)		■	■		■		◆
Coronary artery disease			◯				◆
Diabetes (IDDM)			■		■		◆
Diabetes (NIDDM)			■		■		◆
Liver disease			◯				◆
Malignancy Status	■		■	■	■	■	◆
Peripheral vascular disease			◯				◆
AIDS			◯				■
Stroke			◯				◆
Dementia			◯				
Hemiplegia			◯				◆
Connective tissue disease			◯				◆
Leukemia/Lymphoma			◯				◆
Peptic ulcer disease			◯				◆
ECG	■					■	
Temperature		◯					
Blood Pressure	■	■				■	
pH		■					
Pulse Rate	■	■				■	
Haemoglobin	■					■	
Hematocrit		■					
WBC	■	■				■	
Urea	■					■	
Sodium	■	■				■	
Potassium	■	■				■	
Creatinine		■				■	
GCS	■	■				■	
Blood Loss	■					■	
Peritoneal contamination	■					■	
PaO2		◯					
A-a Gradient		◯					
Steroid chronic use					◯		
Ascites					◯		
Ventilator Dependent					◯		

The aim of this study was to assess surgical mortality and generate a peri-operatory score system, SMATT score (Surgical Mortality Assessment & Stratification) pre-operative risk estimation using readily available and multidimensional patients, and structural characteristics in elective and emergency surgical procedure.

## Methods

### Study design and patient population

We carried a retrospective analysis of prospectively collected data from a large cohort of patients undergoing surgery in our Institution from 2002 to 2014. The study endpoint was perioperative hospital 30 days mortality for any cause following one or more surgical procedures. The inclusion criteria were: a) age 16 or more; b) surgery in one of the surgical wards of our hospital: cardiac, general and vascular surgery, neurosurgery, gynecology, obstetrics, ear-nose-throat surgery, urology, ophthalmology, and orthopedics.

The final aim was the generation of a risk scoring system (SMATT score) based on the analysis of pre-operative data predicting mortality in elective and emergency patient.

All patients included in the present study have signed the consent for using individuals data.

### Data source

Data were retrieved from the 3 electronic registries: 1) the overall administrative database, where data pertaining to the hospital discharge form, including patients demographics, clinical data and procedures that were coded according to the ICD9-CM (International Classification of Diseases, 9th revision - Clinical Modification) are stored; 2) the operating theatre registry that collects data pertaining to the surgical act; 3) the anesthesiology registry with data regarding preoperative, intra- and perioperative clinical assessment of the patient^19^. Further perioperative deaths occurring after discharge but within 30 days were retrieved from the regional administrative databases from Italian Heath system.

Planned surgical complexity was defined according to NHS guidelines Preoperative Tests^20^; anesthesiology complexity was graded on the basis of the ASA physical status classification system^21^. The database structure and relationships are presented in the Supplemental Digital Content 2. As a single patient could have had repeated hospitalizations and more than one surgical procedure, each one of the latter was identified by a progressive number within a hospitalization nosology number and a patient’s unique personal identifier.

### Statistical analysis

All statistical analyses were performed using STATA 13.1 statistical software (Stata Corporation; College Station, TX, USA). The level of significance was set at the two-tailed P-value <0.05. The Bonferroni correction was used for post-hoc comparisons within multiple categories variables.

Mean and standard deviation (SD), or median and 25th-75th percentiles were used to describe continuous variables, count, and percent for categorical variables. Perioperative mortality was reported with exact binomial 95% confidence interval (95%CI). For the purpose of the analysis, age was categorized into 4 groups and BMI into 3 groups. Comorbidities were considered both by type and after grouping by apparatus. A series of candidate predictors for mortality (Table 2) were assessed by means of univariable logistic models. The odds ratio (OR) and 95%CI was computed to measure the strength of the association. Given the large number of variables assessed, a consensus between the authors, including surgeons, hygiene, and public health specialists, bioengineer and biostatistician identified clinically meaningful candidate predictors (Table 3) to be included in a multivariable logistic model. The absence of collinearity between predictors was assessed before fitting the multivariable model. The statistical unit was hospitalization; thus Huber-White robust standard errors were computed while clustering on patients to account for the lack of independence of measures. Model discrimination was assessed with the area under the model Area Under the Curve-Reciever Operating Characteristic (AUC-ROC). Ten-fold cross-validation was performed to validate the model, and the discrimination AUC-ROC statistic was recalculated for confirmation. Finally, the linear predictor (or prognostic score) was calculated as the linear combination of the regression coefficients with the values of the co-variables for each patient in the dataset. For an easier calculation of the score, we multiplied each regression coefficient by 100 to obtain integers and built the score to a range from 0 to 100 using normalization. A new logistic model was then fitted, with the prognostic score as the sole predictor and model % of correctly predicted cases together with computed predictive values. Probabilities of death given the vector of predictors were derived from the model and plotted against the score.

**Table 2:** Univariable analysis.

	N° hospitalizations	N° deceased	% deaths	Odds ratio	95% CI	P value
Patient related variables						
Gender						**<0.001**
• *Male*	*75,942*	1*,825*	*2.40*	*1.68*	*1.56–1.80*	*<0.001*
• *Female*	*88,211*	*1,274*	*1.44*	*1*	—	—
Age Classes						**<0.001**
• *16–30*	*20,854*	59	*0.28*	1	—	—
• *31–50*	*49,371*	216	*0.44*	*1.55*	*1.16–2.01*	*0.003*
• *51–70*	*55,376*	929	*1.68*	*6.01*	*4.62–7.83*	<*0.001*
• >71	*38,552*	*1,895*	*4.92*	*18.22*	*14.02–23.67*	<*0.001*
BMI Classes						**<0.001**
• *Underweight*	*6,630*	247	*3.73*	1	—	—
• *Normal over-weight*	*135,695*	*2,542*	*1.87*	*0.49*	*0.43–0.56*	<*0.001*
• *Obesity*	*21,828*	310	1*.42*	*0.37*	*0.31–0.44*	<*0.001*
ASA Score						**<0.001**
• 1	62,368	31	0.05	1	—	—
• 2	73,237	497	0.68	13.74	9.55–19.76	<0.001
• 3	24,963	1,512	6.06	129.65	90.12–186.53	<0.001
• 4	3,038	799	26.30	717.59	473.68–1,087	<0.001
• 5	348	254	72.99	5,434	2,655–11,120	<0.001
Surgeries in previous 10 years (N°)						**0.06**
• 0	120,253	2,324	1.93	1	—	—
• 1–5	42,497	748	1.76	0.91	0.84–0.99	0.025
• 6–10	1,042	21	2.02	1.04	0.68–1.61	0.85
• ≥11	361	6	1.66	0.86	0.38–1.92	0.71
Cardiovascular Disease						**<0.001**
No	97,680	809	0.83	1	—	—
Yes	66,473	2,290	3.45	4.27	3.94–4.63	<0.001
Respiratory Disease						**<0.001**
*No*	*143,079*	*2,347*	*1.64*	*1*	—	
*Yes*	*21,074*	752	*3.57*	*2.22*	*2.04–2.4*1	*<0.001*
Hepatic- Renal Disease						**<0.001**
*No*	*143,003*	*2,346*	*1.64*	*1*	—	
*Yes*	*21,150*	753	*3.56*	*2.2*1	*2.04–2.41*	*<0.001*
Neurological disease						<0.001
*No*	*145,979*	*2,279*	*1.56*	*1*	—	
*Yes*	*18,174*	820	*4.5*1	*2.98*	*2.75–3.23*	*<0.001*
Endocrinological/ Metabolic/Hematic disease						**<0.001**
*No*	*133,717*	*2,174*	*1.63*	*1*	—	
*Yes*	*30,436*	925	*3.04*	1*.90*	*1.75–2.05*	*<0.001*
Gastrointestinal						**0.003**
*No*	*136,236*	*2,534*	*1.86*	*1*	—	
*Yes*	*27,917*	565	*2.02*	*1.09*	*0.99–1.19*	*0.067*
Allergies/drug intolerances						**<0.001**
*No*	*1,161*	110	*9.47*	1	—	
*Yes*	1*62,992*	*2,989*	*1.83*	*0.18*	*0.15–0.22*	*<0.001*
Solid Cancer						<0.001
*No*	*153,148*	*2,629*	*1.72*	*1*	—	
*Yes*	*11,005*	470	*4.27*	*2.55*	*2.3*1*–2.82*	*<0.001*
AIDS						**0.55**
*No*	*163,520*	*3,085*	*1.89*	*1*	—	
*Yes*	633	14	*2.2*1	*1.18*	*0.70–1.99*	*0.55*
Traumatic body injury						**0.47**
*No*	*162,658*	*3,067*	*1.89*	*1*	—	
*Yes*	*1,495*	32	*2*.1*4*	*1.14*	*0.80–1.62*	*0.47*
Rheumatologic/autoimmune diseases						**0.01**
*No*	*1*61*,804*	*3,038*	1*.88*	*1*	—	
*Yes*	*2,349*	*61*	*2.60*	*1.39*	*1.08–1.80*	*0.01*
Pancreatic disease (excluding cancer)						**0.36**
*No*	*163,799*	*3,090*	*1.89*	*1*	—	
*Yes*	354	9	*2.54*	*1.36*	*0.71–2.60*	*0.36*
Surgery related variables						
Department						**<0.001**
• *Cardiac surgery*	*10,181*	815	*8.0*1	*1*	—	—
• *General surgery*	*32,677*	909	*2.78*	*0.33*	*0.30–0.36*	<*0.001*
• *Vascular surgery*	*10,643*	430	*4.04*	*0.48*	*0.43–0.55*	<*0.001*
• *Neural surgery*	*9,094*	375	*4.12*	*0.49*	*0.44–0.56*	<*0.001*
• *Gynecology*	*23,362*	17	*0.07*	*0.01*	*0.01–0.01*	<*0.001*
• *Obstetrics*	*7,160*	4	*0.06*	*0.01*	*0.00–0.02*	<*0.001*
• *Ophthalmology*	3*,437*	*3*	*0.09*	*0.01*	*0.00–0.03*	<*0.001*
• *Orthopedics*	*42,209*	341	*0.81*	*0.09*	*0.08–0.11*	<*0.001*
• *Urology*	*10,932*	113	*1.03*	*0.12*	*0.10–0.15*	<*0.001*
• *ENT*	*14,458*	92	*0.64*	*0.07*	*0.06–0.09*	<*0.00*1
Planned surgical complexity						**<0.001**
• *1*	*13,484*	49	*0.36*	1	—	—
• 2	*72,223*	277	*0*.3*8*	*1.06*	*0.78–1.43*	*0.73*
• *3*	*41,402*	811	*1.96*	*5*.4*8*	*4*.1*0–7.32*	<*0.001*
• *4*	*35,931*	*1,863*	*5.18*	*14.99*	*11.26–19.96*	<*0.001*
Surgical procedure priority						**<0.001**
• *Elective*	*136,065*	*1,231*	*0.90*	*1*	**—**	**—**
• *Urgent*	*13,164*	569	*4.32*	*4.95*	*4.47–5.48*	<*0.001*
• *Urgent off-time*	*14,085*	*1,105*	*7.85*	*9.32*	*8.57–10.14*	<*0.001*
• *Emergency*	827	193	*23.34*	*33.34*	*28.04–39.65*	<*0.001*
Previous surgeries in the same hospitalization occasion (N°)						**<0.001**
• 1	158,198	2,262	1.43	1	—	—
• 2–3	5,574	734	13.17	10.45	9.56–11.43	<0.001
• ≥4	381	103	27.03	25.54	20.27–32.18	<0.001

**Table 3:** Multivariable Model, Number of obs = 171060; Wald chi2(42)= 7075.55 Prob> chi2 = <0.001 Model discrimination (10-fold cross-validation Model AUC-ROC) = 0.941, 95%CI 0.939–0.944.

	Odds ratio	CI 95%	P value
Patient related variables			
Gender			**0.50**
• Male	1	—	—
• Female	1.04	0.93–1.15	0.50
Age Classes			**<0.001**
• 16–30	1	—	—
• 31–50	1.55	1.02–2.36	0.04
• 51–70	3.18	2.13–4.75	<0.001
• >71	6.57	4.39–9.81	<0.001
BMI Classes			**<0.001**
• Underweight	1.96	1.64–2.33	<0.001
• Normal over-weight	1	—	—
• Obese	0.88	0.76–1.02	0.1
ASA Score			**<0.001**
• 1	1	—	—
• 2	4.14	2.78–6.18	<0.001
• 3	17.25	11.52–25.83	<0.001
• 4	52.49	34.55–79.74	<0.001
• 5	325	203–521	<0.001
Surgeries in the past 10 years (N°)			**0.43**
• 0	1	—	—
• 1–5	1.00	0.89–1.11	0.88
• 6–10	0.62	0.35–1.11	0.11
• ≥11	0.76	0.29–2.05	0.59
Surgery related variables			
Department			**<0.001**
• Cardiac surgery	1	—	—
• General surgery	1.36	1.17–1.58	<0.001
• Vascular surgery	0.67	0.56–0.80	<0.001
• Neural surgery	0.75	0.63–0.91	0.003
• Gynecology	0.16	0.09–0.28	<0.001
• Obstetrics	0.10	0.04–0.27	<0.001
• Ophthalmology	0.14	0.04–0.43	0.001
• Orthopedics	0.47	0.39–0.56	<0.001
• Urology	0.67	0.52–0.86	0.002
• ENT	0.46	0.35–062	<0.001
Planned surgical complexity SCCI			**<0.001**
• 1	1	—	—
• 2	1.13	0.81–1.57	0.48
• 3	2.34	1.70–3.22	<0.001
• 4	2.33	1.68–3.22	<0.001
Surgical procedure priority			**<0.001**
• Elective	1	—	—
• Urgent	2.85	2.56–3.18	<0.001
• Urgent off-time	3.01	2.73–3.31	<0.001
• Emergency	3.67	2.87–4.70	<0.001
Previous surgeries in the same hospitalization occasion (N°)			**<0.001**
• 1	1	—	
• 2–3	2.94	2.63–3.29	<0.001
• ≥4	6.70	4.91–9.13	<0.001
Cardiovascular Disease	0.93	0.82–1.05	**0.24**
Respiratory Disease	1.09	0.97–1.22	**0.15**
Hepatic- Renal Disease	1.29	1.15–1.44	**<0.001**
Neurological disease	1.17	1.05–1.31	**0.005**
Endocrinologic/ Metabolic/Hematologic disorders	1.11	1.00–1.23	**0.05**
Gastrointestinal disease	0.88	0.78–1.00	**0.03**
Unknown Drug intolerance	0.55	0.42–0.73	**<0.001**
Solid Cancer	1.37	1.20–1.57	**<0.001**
AIDS	2.15	1.14–4.12	**0.02**
Traumatic body injury	1.06	0.70–1.61	**0.77**
Rheumatologic disease	1.00	0.73–1.38	**0.98**
Pancreatic disease	0.67	0.23–2.00	**0.48**

*SCCI = surgical complexity classification index.

## Results

### Study population and outcome

During the study, 121.290 patients underwent 173.017 surgeries in 164.153 admissions. The mean age at admission was 54 years (SD ± 19); 46% were male. The mean BMI was 25 kg/m^2^ (SD 4): 83% of patients were normal or over-weight, 4% underweight and 13% of patients were obese. Patient hospital mortality was 2.56% (95%CI 2.47% to 2.64–3,099 patients), with a mortality of 1.89% (95%CI 1.82–1.95%) over all the 164,153 admissions.

### Correlates of mortality (univariable analysis)

Results of the univariable analyses are provided in Table 2. Most of the tested predictors were associated significantly with in-hospital death following surgery. Mortality was almost twice higher in males than in females; it increased with age and ASA score, but decreased over BMI classes. The presence of comorbidities increased the risk of death: overall, the risk was the highest in the presence of cardiovascular diseases (fourfold increase) and neurological diseases (threefold increase). Specifically, patients with heart failure, pulmonary hypertension, acute kidney injury, cirrhosis or coma showed the highest mortality, with a 6- to a 21-fold increase in risk, while patients with known drug intolerance demonstrated lower mortality (see table, Supplemental Digital Content 3). When considering surgery-related variables, mortality was the highest (8%) for cardiac surgery, followed by neurosurgery (4.1%), whereas it was lower (0.1%) for gynecology, obstetrics, and ophthalmology. Finally, the risk significantly increased with planned surgical complexity (grades 3: 2% and grade 4: 5%), procedure priority (emergency: 23.3%, urgent: 4.3%, urgent-off-time: 7.8%) and the number of re-do surgeries during the same admission ≥2 (from 10.4% to 25.5%).

### Multivariable analysis and derivation of the prognostic SMATT score

**Figure 1:**
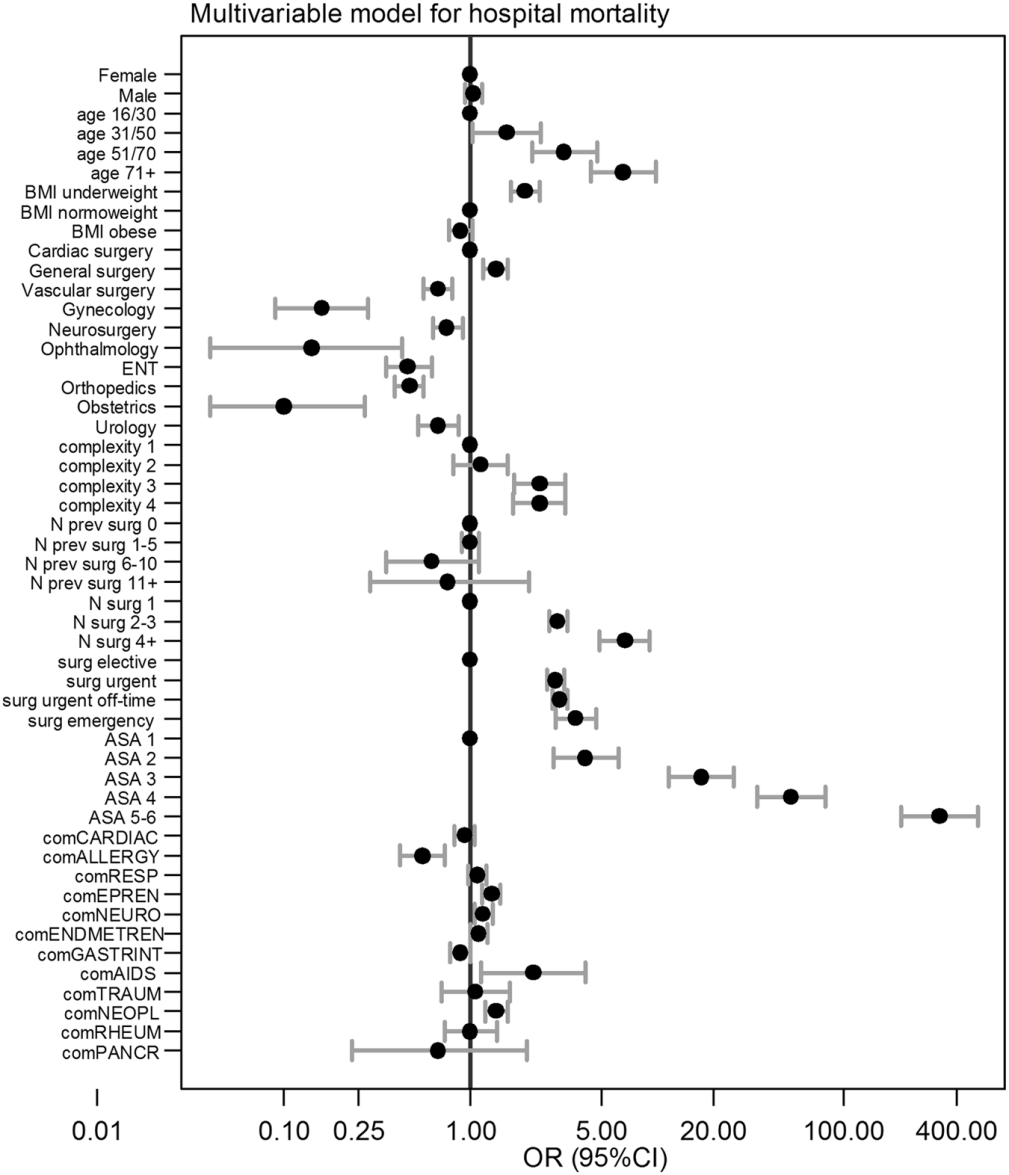
Forrest plot derived from the logistic multivariable model. The estimated odds ratios (OR) are shown as dots, and the 95% confidence intervals as whiskers. The reference category is shown with a dot only on the no effect line (OR = 1). Whiskers to the right of this line correspond to an increased risk of death, with respect to the reference category; whiskers to the left, correspond to a lesser risk than the reference category; whiskers crossing the no effect line denote lack of statistical significance.

The results of multivariable model are detailed in Table 3 and illustrated in Fig. 1. Age, BMI, ASA score, department, planned surgical complexity, surgical priority, previous surgeries in the same hospitalization, cardiovascular, pulmonary, hepato-renal comorbidities, known drug intolerance, cancer, and AIDS were independently associated with mortality after surgery. Model discrimination was optimal (AUC-ROC 0.940, 95%CI 0.938–0.943) and was confirmed after 10-fold cross-validation (AUC-ROC 0.941, 95%CI 0.9439–0.944). The prognostic score was then calculated from the estimated coefficient in the multivariable model, as described in the statistical analysis section. The final score ranged from 0 to 100. The SMATT score confirmed an excellent discrimination ability at 10-fold cross-validation (AUC-ROC 0.945, 95%CI 0.941–0.948). It was able to correctly classify 98.5% of admissions as having a probability of death>50%. The positive and negative predictive values, given the observed mortality rate of 1.89% of all admissions, were 61.4% and 97.99%, respectively.

**Figure 2:**
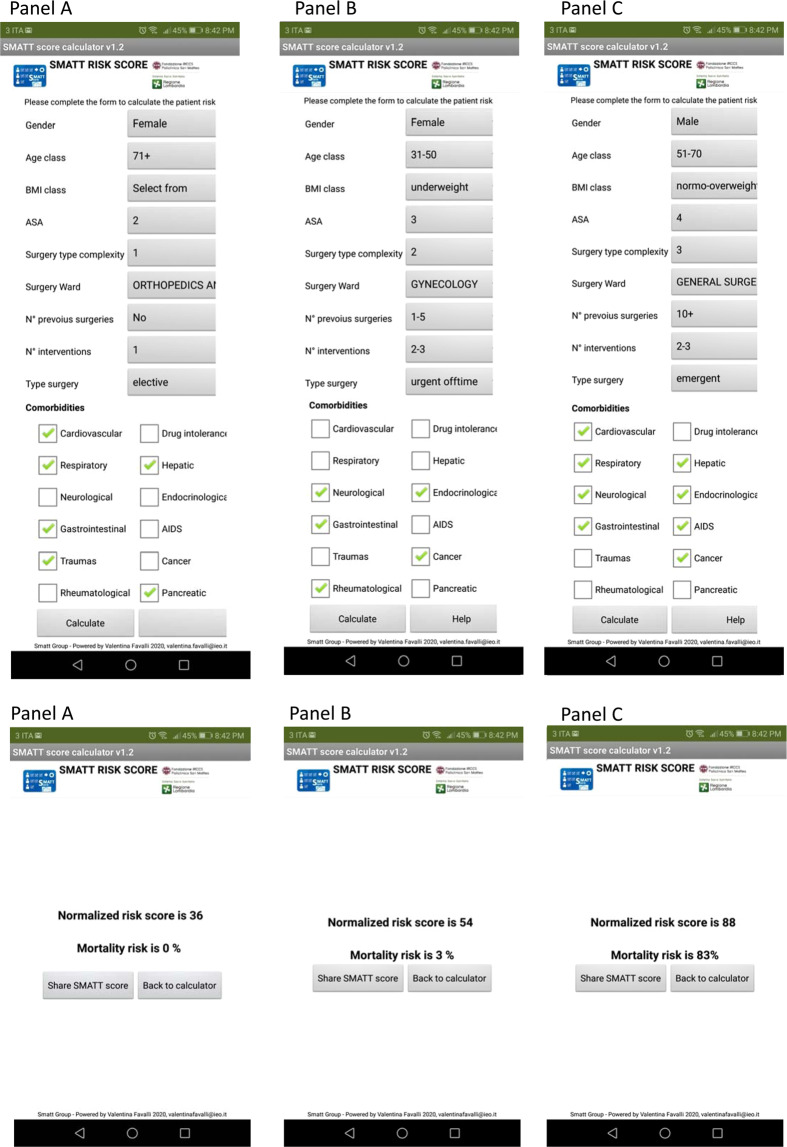
SMATT calculation and probability of death is obtained by the implementation of individual data in the app. Two hypothetical scenarios are shown, with the SMATT score calculated through the app. The derived probability of perioperative death is 0% in panel A (SMATT = 36); 3% (SMATT = 54) in panel B and 83% (SMATT = 88) in Panel C. Panel A). The patient is a 71-years-old woman, with a prior history of ischemic cardiomyopathy and COPD, chronic pancreatitis, peptic ulcer. She was hospitalized for a femoral fracture. Panel B). The patient is a 45-years-old woman, underweight, with a prior history of chronic gastritis, breast cancer, peripheral nervous system disease, osteoarthritis. She has an ASA score 3, she was hospitalized for an elective hysteroannessiectomy. Panel C). The patient is a 54-years-old man, normo-overweight, with a prior history of ischemic cardiomyopathy and COPD, cirrhosis, esophageal varices, AIDS, colon cancer, hyperparathyroidism. He has an ASA score 4, he was hospitalized for an emergency laparotomy for an intestinal obstruction.

Based on these results, we created a dedicated app to assist physicians in calculating the SMATT score at bedside and yielding the probability of perioperative death, together with a nomogram, providing graphical assistance to estimate the probability of death. The app is available on Google Play at following link https://play.google.com/store/apps/details?id=appinventor.ai_valentinafavalli.SMATT. Figure 2 shows the SMATT score calculated with the app together with the corresponding probability of perioperative death in three simulated patients with low, intermediate and high risk (panel A, B and C, respectively). As illustrated in the accompanying nomogram, the probability of death if minimal for SMATT scores up to 50; it increases steadily and linearly for scores above 50, and up to more than 95% for SMATT = 100.

## Discussion

This large surgical cohort allowed us to build a prognostic score (Surgical Mortality Assessment & sTraTification - SMATT score) to be computed preoperatively at bedside, in order to stratify elective and emergency patients according to their risk of perioperative mortality. SMATT showed excellent discrimination and particularly, given the high negative predictive value, it was able to identify low risk patients who possibly would not require high intensity post-operative care. SMATT applies for surgical risk regardless the kind of surgery integrating also multiple patient’s clinical features. SMATT is easy to use, given the proposed associated app, which can be downloaded on a hand held device. Some concern may arise from the low positive predictive value of the SMATT score, making the identification of patients with higher risk more difficult. This issue is, however, shared by all risk scores in the field, given the low rates of perioperative mortality in Western Countries.

Age, BMI, ASA score, surgical specialty, planned surgical complexity, priority (elective, urgent and emergency) and previous surgeries during the same hospital admission, cardiovascular, pulmonary, hepato-renal comorbidities, unknown drug intolerance, cancer and AIDS were independently associated with mortality after surgery. Interestingly, whereas factors such as age and BMI were expected to be associated with surgical mortality^22–26^, our results confirm previous reports, showing that obesity was not a predictive factor for postoperative mortality, while underweight patients demonstrated a higher risk of mortality^27–29^. This is agreeing with those studies: a recent cohort study, including 401227 adult patients (National Adult Cardiac Surgery registry for all cardiac surgical procedures; April 2002- March 2013) and a meta-analysis through June 2015, including 557720 patients, showing that obesity was associated with lower risks after cardiac surgery^30^.

Although a number of risk-scoring systems have already been published^12–18^, most of them were related to specific procedures and surgical specialties. The collective assessment of both patient and structural characteristics may help in preoperative stratification of mortality risk that results from a combination of preoperative, intra-operative and postoperative factors, related to the current surgical event. Although each of these factors may have several standardized features and pathways (i.e. pre-operative diagnosis/indication and kind of surgical procedure), the patient’s features may vary widely and thus require a multidimensional evaluation. Also, using SMATT may lead to a physician-patient communication process more effective^31^.

A common issue when presenting a new score is its value in other settings. Our 10-fold cross-validation procedure confirmed the high discrimination ability of SMATT. Its extensive use in similar or different hospital settings needs further confirmation on independent case series. Given the results of our validation through simulation, we are confident that the prediction of perioperative mortality can be applied in multi-specialty hospitals, with a similar case-mix index as ours of 1.52, and an overall high complexity of organization and care^20^.

This is a retrospective single center study, however, a prospective data collection in three dedicated electronic registries for the administrative, anesthesiology and surgical data, has been systematically implemented in our institution since 2002, offering sufficient and exhaustive information on each single patient. Data analysis did not include costs and budget/resources assigned to the different surgical and anesthesiology teams that may have influenced the outcome. This could have been true in a multi-center study; it is however not the case in a single research hospital such as ours. We are planning to perform a multicentric study to apply the external validation.

We propose the SMATT score as a novel tool for stratification of surgical mortality, amenable for application in large multi-specialty hospitals. Its easy use through a hand-held device will allow a personalized scoring of risk at the pre-operative assessment, based on a multi-dimensional approach. Benchmarking evaluation could help further validate and confirm its potential role and easy application.

### Ethics approval and consent to participate

All patient submit authorization for the processing of personal and sensitive data according to the local procedure approved by ethical board of IRCCS Policlinico San Matteo Foundation, Pavia, Italy.

All patients provided ‘informed consent’ for the use of their data in the present study.

The study design was approved by the Ethics Committee of IRCCS Fondazione Policlinico San Matteo with a ref. number 027/2017.

### Consent for Publication

All the personal data included in the present work have the consent for publication.

### Permission to use the SMATT score logos

We obtain permission to use SMATT score logos in Fig. 2 from Fondazione IRCCS Policlinico San Matteo, the logos do not have any copyright.

## Supplementary information

Risk scores compare.

Database structure and relationships.

Comorbidities.

## Data Availability

All data generated or analysed during this study are included in this published article and its supplementary files.
