# Supplementary material for: A Multidimensional Approach of Surgical Mortality Assessment and Stratification (Smatt Score)
Source: Sci Rep. 2020 Jul 3;10:10964. doi: 10.1038/s41598-020-67164-6 (PMC7335058; doi:10.1038/s41598-020-67164-6)
Supplement: Supplementary file 1 — Risk scores compare. [file 41598_2020_67164_MOESM1_ESM.docx]

**A MULTIDIMENSIONAL APPROACH OF SURGICAL MORTALITY ASSESSMENT AND STRATIFICATION** (**SMATT** **scorE)**

Sara Cutti^1^ M.D., Catherine Klersy^2^ M.D., Valentina Favalli^3^ PhD., Lorenzo Cobianchi^4^ M.D., Alba Muzzi^1^ M.D., Marco Rettani^1^ PhD, Guido Tavazzi^5,6^ M.D. PhD, Maria Paola Delmonte^6^ M.D., Andrea Peloso^4^ M.D., Eloisa Arbustini^3^ M.D., Carlo Marena^1^ M.D.

^1^ Medical Direction, ^2^Service of Clinical Epidemiology & Biometry, ^3^Transplant Research Area, ^4^General Surgery, ^5^ University of Pavia, Department of Clinical, Surgical, Diagnostic and Pediatric Sciences; 6. Department of Anesthesia and Intensive Care,

Foundation IRCCS San Matteo Hospital, Viale Golgi 19, 27100 Pavia, Italy.

***Corresponding Author:***

Carlo Marena, M.D.

Viale Golgi 19,27100 Pavia, Italy

Email: [cmarena@smatteo.pv.it](mailto:cmarena@smatteo.pv.it)

Phone: 0039.0382.503419

***Abbreviated Title:*** *Mortality in surgical patients*

**SUPPLEMENTAL DIGITAL CONTENT 1**

Factors included in the different risk scores:

◼factor shared in the different scores; ◼ uniquely present; ◆ included in comorbidities

| **Mortality Score** | **POSSUM** | **APACHE II** | **Charlson** | **SORT** | **SRS -NSQIP** | **NELA** | **SMATT** |
| --- | --- | --- | --- | --- | --- | --- | --- |
| Age | ◼ | ◼ | ◼ | ◼ | ◼ | ◼ | ◼ |
| Gender |  |  |  |  | ◼ | ◼ | ◼ |
| Present smoker |  |  |  |  | ◼ |  |  |
| Functional Status |  |  |  |  | ◼ |  |  |
| BMI |  |  |  |  | ◼ |  | ◼ |
| Surgical procedure |  |  |  | ◼ | ◼ |  |  |
| Grade of surgery | ◼ |  |  | ◼ |  | ◼ | ◼ |
| N° of procedure | ◼ |  |  |  |  | ◼ | ◼ |
| Urgency of surgery | ◼ |  |  | ◼ | ◼ | ◼ | ◼ |
| Surgical specialty |  |  |  |  |  |  | ◼ |
| ASA score |  |  |  | ◼ | ◼ | ◼ | ◼ |
| Cirrhosis |  | ◼ |  |  |  |  | ◆ |
| Heart failure | ◼ | ◼ |  |  | ◼ | ◼ | ◆ |
| COPD | ◼ | ◼ | ◼ |  | ◼ | ◼ | ◆ |
| Hypertension |  |  |  |  | ◼ |  | ◆ |
| Acute Renal Failure |  | ◼ |  |  | ◼ |  | ◆ |
| Chronic Renal Failure (dialysis) |  | ◼ | ◼ |  | ◼ |  | ◆ |
| Coronary artery disease |  |  | ◼ |  |  |  | ◆ |
| Diabetes (IDDM) |  |  | ◼ |  | ◼ |  | ◆ |
| Diabetes (NIDDM) |  |  | ◼ |  | ◼ |  | ◆ |
| Liver disease |  |  | ◼ |  |  |  | ◆ |
| Malignancy Status | ◼ |  | ◼ | ◼ | ◼ | ◼ | ◆ |
| Peripheral vascular disease |  |  | ◼ |  |  |  | ◆ |
| AIDS |  |  | ◼ |  |  |  | ◼ |
| Stroke |  |  | ◼ |  |  |  | ◆ |
| Dementia |  |  | ◼ |  |  |  |  |
| Hemiplegia |  |  | ◼ |  |  |  | ◆ |
| Connective tissue disease |  |  | ◼ |  |  |  | ◆ |
| Leukemia/Lymphoma |  |  | ◼ |  |  |  | ◆ |
| Peptic ulcer disease |  |  | ◼ |  |  |  | ◆ |
| ECG | ◼ |  |  |  |  | ◼ |  |
| Temperature |  | ◼ |  |  |  |  |  |
| Blood Pressure | ◼ | ◼ |  |  |  | ◼ |  |
| pH |  | ◼ |  |  |  |  |  |
| Pulse Rate | ◼ | ◼ |  |  |  | ◼ |  |
| Haemoglobin | ◼ |  |  |  |  | ◼ |  |
| Hematocrit |  | ◼ |  |  |  |  |  |
| WBC | ◼ | ◼ |  |  |  | ◼ |  |
| Urea | ◼ |  |  |  |  | ◼ |  |
| Sodium | ◼ | ◼ |  |  |  | ◼ |  |
| Potassium | ◼ | ◼ |  |  |  | ◼ |  |
| Creatinine |  | ◼ |  |  |  | ◼ |  |
| GCS | ◼ | ◼ |  |  |  | ◼ |  |
| Blood Loss | ◼ |  |  |  |  | ◼ |  |
| Peritoneal contamination | ◼ |  |  |  |  | ◼ |  |
| PaO2 |  | ◼ |  |  |  |  |  |
| A-a Gradient |  | ◼ |  |  |  |  |  |
| Steroid chronic use |  |  |  |  | ◼ |  |  |
| Ascites |  |  |  |  | ◼ |  |  |
| Ventilator Dependent |  |  |  |  | ◼ |  |  |
