# Supplementary material for: A Multidimensional Approach of Surgical Mortality Assessment and Stratification (Smatt Score)
Source: Sci Rep. 2020 Jul 3;10:10964. doi: 10.1038/s41598-020-67164-6 (PMC7335058; doi:10.1038/s41598-020-67164-6)
Supplement: Supplementary file 2 — Database structure and relationships. [file 41598_2020_67164_MOESM2_ESM.pdf]

A MULTIDIMENSIONAL APPROACH OF SURGICAL MORTALITY ASSESSMENT AND STRATIFICATION (SMATT SCORE)

Sara Cutti[1] M.D., Catherine Klersy[2] M.D., Valentina Favalli[3] PhD., Lorenzo Cobianchi[4] M.D., Alba Muzzi[1] M.D., Marco Rettani[1] PhD, Guido Tavazzi[5,6] M.D. PhD, Maria Paola Delmonte[6] M.D., Andrea Peloso[4] M.D., Eloisa Arbustini[3] M.D., Carlo Marena[1] M.D.

[1] Medical Direction, [2]Service of Clinical Epidemiology & Biometry, [3]Transplant Research Area, [4]General Surgery, [5] University of Pavia, Department of Clinical, Surgical, Diagnostic and Pediatric Sciences; 6. Department of Anesthesia and Intensive Care,

Foundation IRCCS San Matteo Hospital, Viale Golgi 19, 27100 Pavia, Italy.

Corresponding Author:

Carlo Marena, M.D.
Viale Golgi 19,27100 Pavia, Italy
Email: cmarena@smatteo.pv.it
Phone: 0039.0382.503419

Abbreviated Title: *Mortality in surgical patients*

SUPPLEMENTAL DIGITAL CONTENT 2: Database structure and relationships.

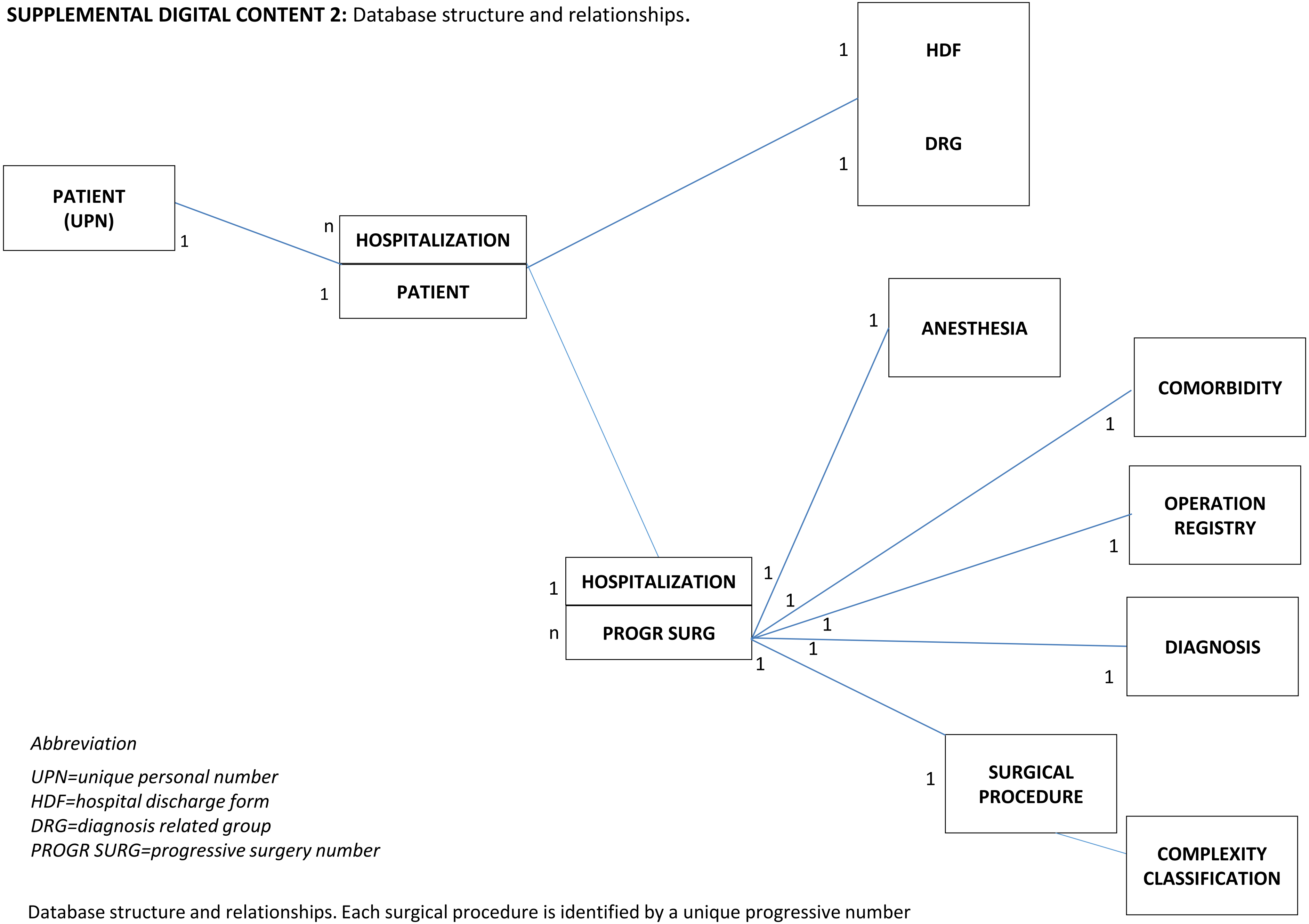

Abbreviation

UPN=unique personal number
HDF=hospital discharge form
DRG=diagnosis related group
PROGR SURG=progressive surgery number

Database structure and relationships. Each surgical procedure is identified by a unique progressive number within a hospitalization nosology number and a unique personal identifier for each patient.
