# Supplementary material for: A Multidimensional Approach of Surgical Mortality Assessment and Stratification (Smatt Score)
Source: Sci Rep. 2020 Jul 3;10:10964. doi: 10.1038/s41598-020-67164-6 (PMC7335058; doi:10.1038/s41598-020-67164-6)
Supplement: Supplementary file 3 — Comorbidities. [file 41598_2020_67164_MOESM3_ESM.docx]

**A MULTIDIMENSIONAL APPROACH OF SURGICAL MORTALITY ASSESSMENT AND STRATIFICATION** (**SMATT** **scorE)**

Sara Cutti^1^ M.D., Catherine Klersy^2^ M.D., Valentina Favalli^3^ PhD., Lorenzo Cobianchi^4^ M.D., Alba Muzzi^1^ M.D., Marco Rettani^1^ PhD, Guido Tavazzi^5,6^ M.D. PhD, Maria Paola Delmonte^6^ M.D., Andrea Peloso^4^ M.D., Eloisa Arbustini^3^ M.D., Carlo Marena^1^ M.D.

^1^ Medical Direction, ^2^Service of Clinical Epidemiology & Biometry, ^3^Transplant Research Area, ^4^General Surgery, ^5^ University of Pavia, Department of Clinical, Surgical, Diagnostic and Pediatric Sciences; 6. Department of Anesthesia and Intensive Care,

Foundation IRCCS San Matteo Hospital, Viale Golgi 19, 27100 Pavia, Italy.

***Corresponding Author:***

Carlo Marena, M.D.

Viale Golgi 19,27100 Pavia, Italy

Email: [cmarena@smatteo.pv.it](mailto:cmarena@smatteo.pv.it)

Phone: 0039.0382.503419

***Abbreviated Title:*** *Mortality in surgical patients*

**SUPPLEMENTAL DIGITAL CONTENT 3:** List of comorbidities observed in the cohort under investigation

| **Detailed comorbidities** | | | | | | | |
| --- | --- | --- | --- | --- | --- | --- | --- |
| - ***Heart failure*** |  |  |  |  |  | ***<0.001*** |  |
| *No* | *161,335* | *2,724* | *1.69* | *1* | *-* |  |  |
| *Yes* | *2,818* | *375* | *13.31* | *8.94* | *7.97-10.03* | *<0.001* |  |
| - ***Ischemic cardiomyopathy*** |  |  |  |  |  | ***<0.001*** |  |
| *No* | *150,691* | *2,386* | *1.58* | *1* | *-* |  |  |
| *Yes* | *13,462* | *713* | *5.30* | *3.48* | *3.19-3.79* | *<0.001* |  |
| - ***Arrythmias*** |  |  |  |  |  | **<0.001** |  |
| *No* | *153,126* | *2,354* | *1.54* | *1* | *-* |  |  |
| *Yes* | *11,027* | *745* | *6.76* | *4.64* | *4.26-5.05* | *<0.001* |  |
| - ***Cardiomyopathy*** |  |  |  |  |  | **<0.001** |  |
| *No* | *162,256* | *2,932* | *1.81* | *1* | *-* |  |  |
| *Yes* | *1,897* | *167* | *8.80* | *5.25* | *4.46-6.17* | *<0.001* |  |
| - ***Arterial Hypertension*** |  |  |  |  |  | **<0.001** |  |
| *No* | *107,598* | *1,391* | *1.29* | *1* | *-* |  |  |
| *Yes* | *56,555* | *1,708* | *3.02* | *2.38* | *2.21-2.55* | *<0.001* |  |
| - ***Arteriopathies*** |  |  |  |  |  | **<0.001** |  |
| *No* | *155,742* | *2,593* | *1.66* | *1* | *-* |  |  |
| *Yes* | *8,411* | *506* | *6.02* | *3.78* | *3.43-4.17* | *<0.001* |  |
| - ***Valvulopathies*** |  |  |  |  |  | **<0.001** |  |
| *No* | *163,300* | *3,038* | *1.86* | *1* | *-* |  |  |
| *Yes* | *853* | *61* | *7.15* | *4.06* | *3.13-5.28* | *<0.001* |  |
| - ***Chronic Obstructive Pulmonary Disease*** |  |  |  |  |  | **<0.001** |  |
| *No* | *145,166* | *2,469* | *1.70* | *1* | *-* |  |  |
| *Yes* | *18,987* | *630* | *3.32* | *1.98* | *1.81-2.17* | *<0.001* |  |
| - ***Interstitial Lung Disease*** |  |  |  |  |  | **<0.001** |  |
| *No* | *163,521* | *3,051* | *1.87* | *1* | *-* |  |  |
| *Yes* | *632* | *48* | *7.59* | *4.32* | *3.22-5.81* | *<0.001* |  |
| - ***Respiratory infections*** |  |  |  |  |  | **<0.001** |  |
| *No* | *162,838* | *3,054* | *1.88* | *1* | *-* |  |  |
| *Yes* | *1315* | *45* | *3.42* | *1.85* | *1.38-2.50* | *<0.001* |  |
| - ***Pulmonary Hypertension*** |  |  |  |  |  | **<0.001** |  |
| *No* | *163,552* | *3,035* | *1.86* | *1* | *-* |  |  |
| *Yes* | *601* | *64* | *10.65* | *6.30* | *4.86-8.18* | *<0.001* |  |
| - ***Acute Kidney Injury*** |  |  |  |  |  | **<0.001** |  |
| *No* | *163,669* | *3,010* | *1.84* | *1* | *-* |  |  |
| *Yes* | *484* | *89* | *18.39* | *12.03* | *9.53-15.17* | *<0.001* |  |
| - ***Chronic Kidney Injury*** |  |  |  |  |  | **<0.001** |  |
| *No* | *161,615* | *2,869* | *1.78* | *1* | *-* |  |  |
| *Yes* | *2,538* | *230* | *9.06* | *5.51* | *4.79-6.34* | *<0.001* |  |
| - ***Other Renal Disease*** |  |  |  |  |  | **0.94** |  |
| *No* | *163,558* | *3,088* | *1.89* | *1* | *-* |  |  |
| *Yes* | *595* | *11* | *1.85* | *0.98* | *0.54-1.76* | *0.94* |  |
| - ***Chronic Hepatopathy*** |  |  |  |  |  | **<0.001** |  |
| *No* | *155,353* | *2,791* | *1.80* | *1* | *-* |  |  |
| *Yes* | *8,800* | *308* | *3.50* | *1.98* | *1.76-2.23* | *<0.001* |  |
| - ***Cirrhosis*** |  |  |  |  |  | **<0.001** |  |
| *No* | *163,195* | *3,002* | *1.84* | *1* | *-* |  |  |
| *Yes* | *958* | *97* | *10.13* | *6.01* | *4.86-7.44* | *<0.001* |  |
| - ***Biliary tract disease*** |  |  |  |  |  | ***0.10*** |  |
| *No* | *153,942* | *2,928* | *1.90* | *1* | *-* |  |  |
| *Yes* | *10,211* | *171* | *1.67* | *0.88* | *0.75-1.03* | *0.10* |  |
| - ***Cerebrovascular diseases*** |  |  |  |  |  | **<0.001** |  |
| *No* | *158,556* | *2,665* | *1.67* | *1* | *-* |  |  |
| *Yes* | *5,597* | *444* | *7.93* | *5.06* | *4.56-5.61* | *<0.001* |  |
| - ***CNS diseases*** |  |  |  |  |  | **<0.001** |  |
| *No* | *161,777* | *2,948* | *1.82* | *1* | *-* |  |  |
| *Yes* | *2,376* | *151* | *6.36* | *3.66* | *3.09-4.33* | *<0.001* |  |
| - ***PNS diseases*** |  |  |  |  |  | **0.42** |  |
| *No* | *161,983* | *3,053* | *1.88* | *1* | *-* |  |  |
| *Yes* | *2,170* | *46* | *2.12* | *1.13* | *0.84-1.51* | *0.42* |  |
| - ***Traumatic brain injury*** |  |  |  |  |  | **<0.001** |  |
| *No* | *162,700* | *3,031* | *1.86* | *1* | *-* |  |  |
| *Yes* | *1,453* | *68* | *4.68* | *2.59* | *2.02-3.31* | *<0.001* |  |
| - ***Coma*** |  |  |  |  |  | **<0.001** |  |
| *No* | *163,594* | *2,982* | *1.82* | *1* | *-* |  |  |
| *Yes* | *559* | *117* | *20.93* | *14.26* | *11.60-17.53* | *<0.001* |  |
| - ***Epilepsy*** |  |  |  |  |  | **0.32** |  |
| *No* | *163,875* | *3,096* | *1.89* | *1* | *-* |  |  |
| *Yes* | *278* | *3* | *1.08* | *0.57* | *0.19-1.67* | *0.32* |  |
| - ***Depression*** |  |  |  |  |  | **0.90** |  |
| *No* | *156,394* | *2,954* | *1.89* | *1* | *-* |  |  |
| *Yes* | *7,759* | *145* | *1.87* | *0.99* | *0.84-1.17* | *0.90* |  |
| - ***Coagulopathies*** |  |  |  |  |  | **<0.001** |  |
| *No* | *163,116* | *3,057* | *1.87* | *1* | *-* |  |  |
| *Yes* | *1,037* | *42* | *4.05* | *2.21* | *1.62-3.01* | *<0.001* |  |
| - ***Anemia*** |  |  |  |  |  | **<0.001** |  |
| *No* | *159,953* | *2,951* | *1.84* | *1* | *-* |  |  |
| *Yes* | *4,200* | *148* | *3.52* | *1.94* | *1.64-2.30* | *<0.001* |  |
| - ***Blood cancers*** |  |  |  |  |  | **<0.001** |  |
| *No* | *161,935* | *3,020* | *1.86* | *1* | *-* |  |  |
| *Yes* | *2,218* | *79* | *3.56* | *1.94* | *1.55-2.44* | *<0.001* |  |
| - ***Diabetes Insulin Dependent*** |  |  |  |  |  | **<0.001** |  |
| *No* | *161,978* | *2,987* | *1.84* | *1* | *-* |  |  |
| *Yes* | *2,175* | *112* | *5.15* | *2.89* | *2.38-3.51* | *<0.001* |  |
| - ***Diabetes Non-Insulin Dependent*** |  |  |  |  |  | **<0.001** |  |
| *No* | *152,256* | *2,630* | *1.73* | *1* | *-* |  |  |
| *Yes* | *11,897* | *469* | *3.94* | *2.33* | *2.11-2.58* | *<0.001* |  |
| - ***Thyroid disorders*** |  |  |  |  |  | **0.44** |  |
| *No* | *152,345* | *2,887* | *1.90* | *1* | *-* |  |  |
| *Yes* | *11,808* | *212* | *1.80* | *0.95* | *0.82-1.09* | *0.44* |  |
| - ***Enteric diseases*** |  |  |  |  |  | **<0.001** |  |
| *No* | *157,861* | *2,873* | *1.82* | *1* | *-* |  |  |
| *Yes* | *6,292* | *226* | *3.59* | *2.01* | *1.75-2.31* | *<0.001* |  |
| - ***Gastric diseases*** |  |  |  |  |  | **0.007** |  |
| *No* | *140,514* | *2,705* | *1.93* | *1* | *-* |  |  |
| *Yes* | *23,639* | *394* | *1.67* | *0.86* | *0.78-0.96* | *0.007* |  |
| - ***Drugs intolerances (specified)*** |  |  |  |  |  | **<0.001** |  |
| *No* | *139,573* | *2,745* | *1.97* | *1* | *-* |  |  |
| *Yes* | *24,580* | *354* | *1.44* | *0.73* | *0.65-0.81* | *<0.001* |  |
| - ***Adverse reactions to contrast media*** |  |  |  |  |  | **0.08** |  |
| *No* | *162,442* | *3,057* | *1.88* | *1* | *-* |  |  |
| *Yes* | *1,711* | *42* | *2.45* | *1.31* | *0.96-1.78* | *0.08* |  |
| - ***Other allergies (not specified)*** |  |  |  |  |  | **0.37** |  |
| *No* | *26,719* | *486* | *1.82* | *1* | *-* |  |  |
| *Yes* | *137,434* | *2,613* | *1.90* | *1.05* | *0.95-1.15* | *0.37* |  |
